# Supplementary figures and images for: Genetic diversity and evolution of Hantaan virus in China and its neighbors
Source: PLoS Negl Trop Dis. 2020 Aug 20;14(8):e0008090. doi: 10.1371/journal.pntd.0008090 (PMC7462299; doi:10.1371/journal.pntd.0008090)

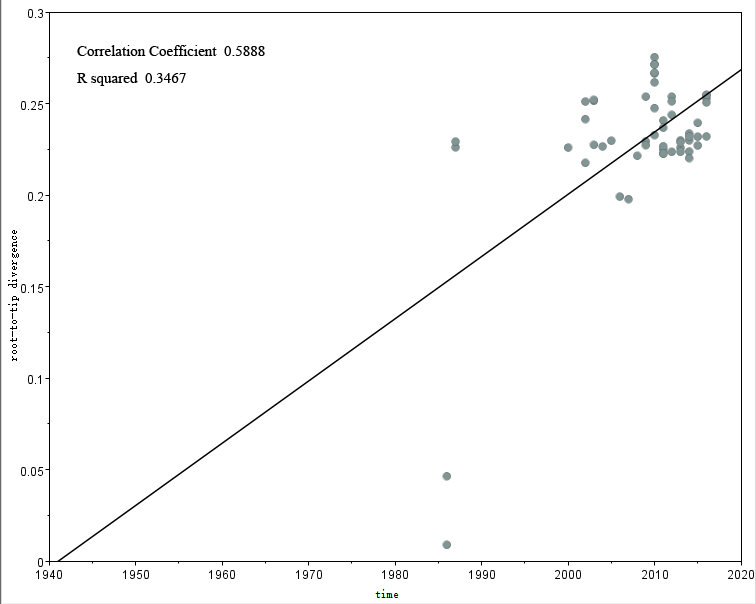

Supplement: S1 Fig — (TIF) [file pntd.0008090.s001.tif]

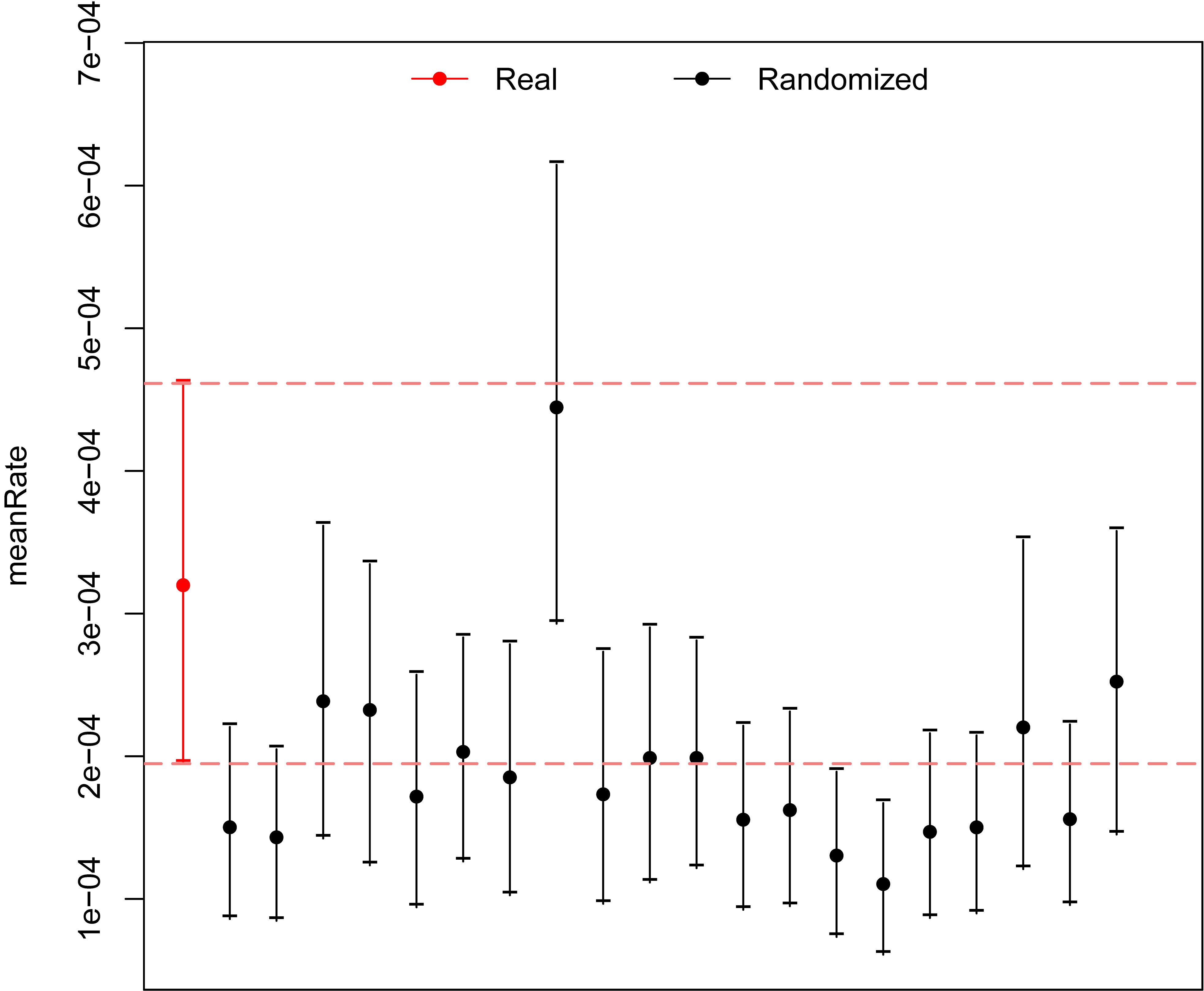

Supplement: S2 Fig — BEAST, Bayesian evolutionary analysis sampling trees; DRT, date-randomization test. (TIF) [file pntd.0008090.s002.tif]
