# Supplementary material for: Genetic diversity and evolution of Hantaan virus in China and its neighbors
Source: PLoS Negl Trop Dis. 2020 Aug 20;14(8):e0008090. doi: 10.1371/journal.pntd.0008090 (PMC7462299; doi:10.1371/journal.pntd.0008090)
Supplement: Attachment — Submitted filename: Response letter[PNTD-D-20-00046].docx [file pntd.0008090.s005.docx]

**Point to point responses**

**Reviewer 1：**

Q1. At line 180 it is noted that “ Only parameter estimates with ESS values exceeding 200 were accepted.” Does this mean that all parameter values had ESSes exceeding 200 or just that those used in the analysis did? Ideally all parameters should have ESSes exceeding that threshold not just those parameters of direct interest, because there is no explicit tracking of the mixing of the tree topology, which often mixes worse that everything else, and most other parameters are themselves conditional on that topology. If a significant number of parameters in the analyses did not have ESSes greater than 200, the analysis should be rerun. If all parameters did have ESSes greater than 200, that statement should be modified for clarity.

A1. Thanks for your advice. As you suggested, it should be all parameters should have ESSes greater than 200. We have corrected the description as ‘The results were accepted only if all the parameters have effective sample sizes over 200’ and ‘Convergence was assessed by estimating the effective sampling dates using Tracer, and accepting ESS values of 200 or more for all the parameters’ in the revised manuscript (*Page 9, Line 164-165; Page 9, Line 170-273).*

Q2. At line 262 it is noted that “The branching order within the lineages differed in S and M trees, which indicates the different evolutionary history between the M and S segments.” It would be useful to add a sentence clarifying if this difference in branching order between the S and M segments is well supported, or whether there is high topological uncertainty in the relevant regions.

A2. Thanks for your suggestion. The branching orders are well supported and we have added the sentence ‘It should be clarified that all the branching orders are well supported’ in the revised MS (*Page 15, Line 248-249).* Also, the Posterior node probabilities and SH-like approximate likelihood values/ultrafast bootstrap values are shown in Figure 2.

Q3. The estimation of the substitution rate in this paper is concerning. The bounds estimated are “a 95% HPD that ranged from 1.0001 х 10-4 to 3.2076 х 10-4. The lower bound is butting right up against the edge of the uniform prior placed on the parameter by the authors, which ends at 1 х 10-4. This is a strong indicator that the data supports a lower evolutionary rate than the prior is allowing. A looser prior (such as a lognormal with 95% of the density lying between 1 х 10-4 and 1 х 10-3) would have avoided this issue, while providing much the same prior information. The model should be rerun with such a looser prior to test the how this changes the estimated evolutionary rate unless the authors have a convincing argument that their assessed subjective probability of the rate being below 1 х 10-4 genuinely is zero (though this seems unlikely).

A3. Thanks for your advice and we re-did the coalescent and evolution analyses with a looser prior, a lognormal with 95% of the density lying between 1 х 10^-4^ and 1 х 10^-3^. We added the description as ‘Time-scale was inferred using an informative substitution rate prior (a lognormal with 95% of the density lying between 1 х 10-4 and 1 х 10-3 substitutions/site/year) previously estimated for the N gene of rodent-borne hantavirus’ in the revised MS *(Page 8, line 157-160).* A more reliable substitution mean rate (2.0185 х 10^-4^ with a 95% HPD that ranged from 9.3435 х 10^-5^ to 3.2011 х 10^-4^) was estimated. So the following work was re-run using this more suitable prior. Except the migration events detected before, a new migration event, from Shaanxi to Hunan, was detected using this looser prior. *(Page 18, Line 285-287; Page 18, Line 294-296; Page19, Line311-312)*

Q4. Table 2 is unclear, as it is not actually stated what the amino acid change is in each epitope. A column showing something along the lines of “V -> I” should be added in order to make the table more user friendly.

A4. Thank you for pointing this out. The substitute sites were marked in bold. We also added a column in Table 3 to show what the amino acid change is. *(Page 16, Line 262)*

Q5. Figure 3 is weirdly stretched and letters have different heights (the “I”s for example are disproportionately tall), this should be amended, so that at least all letters have the same height.

A5. Thank you for pointing this out. We have adjusted the typeface in Figure 3 in order to keep all the letters in same height. *(Page 16, Line 260)*

Q6. It would be useful to change pane B of figure 4 to a map of China with each province coloured by the posterior probability that it contained the root, so that the data can considered spatially, as it is discussed in the text (i.e. to see how highly supported areas are spatially co-located).

A6. Thanks for suggestion. We have made correction according to your comments. We have change Figure 4B to a map colored by the root state posterior probability. *(Page 18, Line 297)*

Q7. At line 379 the authors note: “Markov jump estimates between different locations pinpointed Heilongjiang as an important source of HTNV epidemics in northeast of China and far east of Russia. This finding may explain the high endemic in this area since 1931, when HFRS was first recognized in northeast of China.” It seems more likely that the presence of virus in Heilongjiang allowed it to become the source of the the HTNV epidemics in other regions rather than vice versa. This alternative possibility should be quickly addressed.

A7. Thank you and we are sorry for our inappropriate description. It is exactly what the author wanted to express. But it may be misunderstood because of improper description. We have corrected the sentence as ‘This finding may explain the high endemic in these areas since 1931, when HFRS was first recognized in northeast of China, due to the migration from Heilongjiang to its surroundings’ in *Page 23, Line 382-384.*

Q8. The authors should comment on how much of the estimated recent increase in the population size of the viruses estimated in their skyline analysis they believe is real and how much is due to the sampling of recent diversity being much more complete than historical diversity. If few lineages that were present historically survive, there is limited information to estimate the demography backwards in time, as much of the diversity that was present then is missing from a sample collected in the present era, an issue that may be exacerbated by the auto-correlative effect that is inbuilt in the skyline demographic prior which penalises changes in demography when the evidence for them is weak.

A8. Thanks for question. Just like what you said, uncertainty of demographic analyses increases towards the root of the genealogy, where population history is reconstructed from fewer lineages. And we must admit that majority of samples in our dataset were collected recent years. So the estimated recent increase in the population size of the viruses is not precise. But we think our results may referable because the period of population size’s increasing is in accordance with epidemiology data and can be explained by climate changes. To get a more precise result, we will keep noticing the update of gene databases or collect HTNV samples and sequence by ourselves, in order to gain more available sequences and estimate the demographic history of HTNV. We have discussed about this in the revised MS’s discussion *(Page 27, Line 470-474)*.

Q9. The definition of an acronym should occur with the first use throughout.

A9. Thanks for pointing out. We have corrected the errors in the article.

Q10. The authors should note whether any priors for Bayesian analyses were modified from their default values beyond those mentioned in the text. If not, the statement “All other priors were left on their defaults” would aid in clarity.

A10. Thanks for your advice. Indeed, all other priors in BEAST were left on their defaults. We have clarified it as ‘All other priors were left on their defaults’ in MS’s Method *Page 9, line 163.*

**Reviewer 2：**

Q1. Tests taking strong negative selection into account are needed to properly estimate branch lengths and therefore TMRCA analysis. Additionally, the substitution rate should be validated using a date-randomisation test and the temporal signal of the dataset, which the authors analysed using TempEST, should be shown in order to give an idea of how strong the clock signal is.

A1. Thanks for pointing out. As your suggestion, the selection pressure analyses were conducted using Datamonkey with five varying methods, including single likelihood ancestor counting (SLAC), fixed effects likelihood (FEL), mixed effects model evolution (MEME), fast unconstrained Bayesian approximation (FUBAR) and adaptive Branch-Site Random Effects Likelihood *(Page 7 Line135-143)*. The results showed that purifying selection plays a dominated role in HTNV evolution *(Page 16 Line 263-275)*. So the estimated branch lengths and tMRCA might be underestimated. We have discussed it in *Page 26, Line 452-456*. Furthermore, none of the amino acid substitution sites is being subjected to positive selection.

The root-to-tip analyses using TempEst showed a positive correlation between genetic divergence and sampling time. The result of TempEst has been added in supplementary materials. A date-randomization test has also been conducted as your advice but, unfortunately, our dataset failed to pass the test. As far as we known, the date-randomization test is stricter than root-to-tip analyses and it would be prefect if the dataset can pass the test. The failure may be due to the high similarity of HTNV sequences and unbalanced sampling dates.

In consideration of the positive correlation in root-to-tip analyses, we think our dataset still shows clock signal even it is not such strong. So we hope the results would be referable but can be improved in the future. We will pay close attention to the update of gene databases and collect HTNV samples and sequence by ourselves, in order to gain more available sequences and more precise substitution rate. We have discussed it in revised MS’s discussion (*Page 26, Line 446-452*).

Q2. The authors note that isolates from Guizhou province were highly divergent and group in phylogroups B, C, E, F and I. They do not discuss the possibility of movement of HTNV between areas at this point, and whether it is possible that isolates which evolved in Guizhou province have been dispersed recently into other regions - this would tie together with figure 6 where the movement of HTNV is analysed.

A2. Thanks for your question. It is really true as you suggested that we didn’t deny the possibility of movement of HTNV from Guizhou to other regions. Both the phylogenetic analyses and stat change counts indicated the dispersion from Guizhou to other regions, even though no significant migration was found by phylogeographic inference. We have discussed it as ‘Our Markov jump estimate as well as the phylogenetic correlation with other regions also indicated the dispersion from Guizhou to other areas, even though no significant migration was found by phylogeographic inference. Furthermore, root state posterior probabilities of Guizhou also in a high level. So we can’t deny the possibility of Guizhou to be the place of HTNV common ancestor’ in discussion *(Page 23, Line 397-401)*.

Q3. Fig.1 is difficult to interpret - it may be more effective to convey the isolate sampling locations on a map, with pie charts depicting the years of isolations of samples with the size of the charts directly related to the number of samples from that region. It may also be more effectively communicated as a simple table.

A3. Thanks for pointing out the unclear figure. A map with pie charts depicting the years of isolations of samples were shown in Fig.1. And we have added a table descripting the years and host features of the samples from different regions *(Page 11, Line 197-203)*.

Q4. A map of the sampling locations would also be helpful when interpreting the ML trees presented in Fig.2, to help the reader determine whether geographically distant isolates also appear more or less divergent.

A4. Thanks for the suggestion. The map has been added in Fig1. And the region and host features for each sample were labelled after the strains’ names in Fig2. Also, the abbreviations have been added below the letters identifying the phylogroups to indicate the regions associated to each group *(Page 14, Line 236-240)*.

Q5. Figure 2 is missing a distance bar showing the divergence of the isolates as substitutions/site/year.

Q5. Thank you for pointing it out. The distance bars have been added in Figure2 *(Page 14, Line 236)*.

Q6. Figure 4 is presented well however the colour coding changes from part A to part B making interpretation of the root analysis more difficult.

A6. Thanks for pointing out the unclear description. We have changed Figure 4B to a map colored by the root state posterior probability. The histogram of root was put on part A using the same color with the MCC tree. *(Page 18, Line 297-304)*

Q7. In figure 5 the Bayes factor of the movement may be better presented as a colour gradient as opposed to line width, with a legend clearly showing the gradient - this may be easier to interpret than line width.

A7. Thanks for the valuable suggestion. We have made correction according to the Reviewer's comments. The Bayes factor in Figure 5 has been presented as a color gradient now *(Page 19, Line315-321)*.

**Reviewer 3：**

Q1. The detail of the sequences in the datasets used in the study and presented in the fig 1 as stack columns is very difficult to interpret. It would help having first a panel showing a map of the provinces from east China and neighbouring South Korea and Russia with spot illustrating a range for the number of collected sequences. A table summarizing the data of the number of sequences collected per region, host and years could also be added. Also in the corresponding result section there is no remark made on the distribution of the collected sequences. The authors should comment for instance on the higher number obtained from South Korea then Zhejang, the main hosts and also the few isolates from Rattus norvegicus and possible relationship between HTNV prevalence in the natural reservoir and human host in different regions.

A1. Thanks for the valuable suggestion. We have made correction according to your comments. A map with pie charts depicting the years of isolations of samples were shown in Fig.1 *(Page 11, Line 197-203)*. And we have added a table descripting the years and host features of the samples from different regions *(Page 10, Line 195)*. The features of the dataset were introduced in the revised manuscript, too. Such as ‘Most of the samples were collected during 2001-2005’, ‘More samples were collected from South Korea and Zhejiang, with 67 and 36 strains separately. Furthermore, Guizhou and Shaanxi also had higher number of HTNV samples’ and ‘*Apodemus agrarius,* the main reservoir host for HTNV, has the highest number of samples. A total of 148 (62.2%) strains were isolated from Aa’. *(Page 10, Line 185; Page 10, Line 187-189; Page 10, Line 192-193)*

Q2. The authors eliminated recombination and reassortment events from analysis to reduce bias but the potential impact it could have on the phylogenetic trees is not explained, although such events contribute to the evolution of orthohantaviruses.

A2. Thanks for pointing out. We agree, just as what you said, both recombination and reassortment events play important roles in the evolution of HTNV. We discussed it in manuscript *Page 22, Line 361-374.* But it is reported that recombination will bias the shape of the inferred phylogenetic tree, the branch length and reconstruction of ancestral sequences *(Miguel Arenas and David Posada 2010 10.1534/genetics.109.113423; Mikkel H. Schierup and Jotun Hein 2000 PMCID: PMC1461297; Martin DP et al 2015 10.1093/ve/vev003*). So we didn’t use the recombination isolates for phylogenetic analyses, but which doesn’t mean we deny the contributions of recombination in HTNV evolution. The references and explanations were added in the revised MS *(Page 6, Line 108-111)*.

Q3. The group clustering of S an M sequences together with regions is clear. However, the many different colours used in the Fig.2, make the reading difficult. In addition to the colour legend, the authors should add, into brackets below the letters identifying the phylogroups, right side of the trees, the same abbreviations as used in fig.1, to indicate the regions associated to each group.

A3. Thanks for suggestion. As your advice, the abbreviations have been added below the letters identifying the phylogroups to indicate the regions associated to each group. And the region and host features for each sample were also labelled after the strains’ names in Fig2 *(Page 14, Line 236-240)*. We hope it would be helpful to interpret the phylogenetic trees.

Q4. The identification of 42 specific amino acid substitutions associated to the different groups is interesting. The authors could discuss more the relationship with the immune epitopes: - are they B or T cells epitopes: -the fact that most of the varying sites are found in Gn (26/42), but only 6 are located in a immune epitope in contrast to the 11/42 sites found in Gc among which as much as 10 positions belong to such epitopes; -etc..

A4. Thanks for your valuable advice. Considering your suggestion, we pay more attention on these epitopes. We found 21 T cell epitopes and 3 B cell epitopes totally. And some of the significant epitopes were discussed. More positions in Gc were found may due to the different function of Gn and Gc. Also, it should be mentioned that more epitopes on Gc(82 epitopes) were reported on IEDB than Gn(28 epitopes). Also, two kinds of mutations on two of the reported epitopes (437GQ-RKVILTKLVIGQ451 and 954-LVTKDIDFD-963) indicating the possibility of immune escape were also discussed, too. Furthermore, we investigated the selection pressure and found no positive selection site on these epitopes.

We have discussed these in the revised manuscript (*Page 20, Line 339-353）*.

Q5. The different ML approaches, phylogeographic linkage and state transition analyses lead to an impressive amount of information but still some aspects are quite intriguing and could be worth considering such as: -the origin of the common ancestor in Zhejang in 1198, not appearing as a radiation center, -the high genetic diversity of HTNV from Ghizou segregating in different phylogroups, explained by a long evolution history relating to topology but also a lack of sequences. Could Ghizou be the place of HTNV common ancestor in China. This should be confronted to the study of Zou et al (8) proposing Ghizou as a radiation centre, -the different evolution history of M and S segment also associated to topology is not clear.

A5. Thanks for the valuable suggestion. The origin of HTNV was thought in Zhejiang but no migration from Zhejiang to other regions was found. It may result from the lack of whole-genomic data from Zhejiang and its surrounding areas. We discussed about it in the revised manuscript. (*Page 22, Line 376-379)*

Previous research considers Guizhou as a radiation center according to the topology of phylogenetic tree. In our research, the results of stat change counts indicated the dispersion from Guizhou to other regions, even though no significant migration was found by phylogeographic inference. We recognized Zhejiang as the original of HTNV according to the root state posterior probability, which showed the highest level. Also, the root state posterior probability of Jiangxi and Guizhou showed a high level. So, indeed, it is possible for Guizhou to be the place of HTNV common ancestor in China. But maybe we need more available sequences to get a more precise result. We discussed this in *Page 23, Line 397-401*.

The different of topology shown in S and M phylogenetic tree was also shown in others’ research, but it hasn’t been explained before. We guess it may due to the different evolution history of these two segments. This assumption is identified with Hae Ji Kang et al *(doi: 10.1128/JVI.00285-14)* and Maria Razzauti et al *(doi: 10.1371/journal.pone.0064447)* but need to validated in the future*.* We added the sentences ‘The topologies between the M and S segments are different. We inferred it due to the different evolutionary history, which was in accordance with others’ assumption. But it is still need to be validated in the future’ in the revised manuscript *(Page 21, Line 357-360)*

Q6. Important issues to be addressed are whether and how a low number or incompleteness of sequences, as well as including or not other regions, could impact the results. The case of South Korea and phylogeographic linkage could be further discussed. The authors propose that their findings could help to understand hantavirus epidemics and to develop prevention strategies. They should discuss in which way and include similar recent approaches strengthening this idea (for instance, Zheng et al 2019 doi.org/10.1371/journal.pntd.0007148)

A6. Thanks for questions. As we did the research using whole-genome sequences, partial sequences are not comparable, for instance, partial sequences from different researches may located in different regions of whole genome. So lacking of whole-genome sequences may cover up the diversity of HTNV and the unbalanced sequences quantity form different regions might result in the loss of migration events. Also, the contribution to HTNV evolution and spread may be weaken by low number of available sequences. We discussed these in *Page 27, Line 479-481.*

The characteristics of sequences from South Korea has been described before *(Kim WK et al DOI: 10.1038/srep26017; Kim JA DOI: 10.1371/journal.pntd.0004650)*. So the case of South Korea and phylogeographic linkage between South Korea and Jiangsu was discussed briefly as ‘The samples collected from South Korea have been studied before so we don’t discuss too much here. But the migration of HTNV from South Korea to Jiangsu indicates the possibility of HTNV import from other countries’ in *Page 23, Line 388-390.*

The results showed specific amino acid substitutions on epitopes, which could be connected with different regions. We hope it could be an indicator of immune effect evaluation of vaccine in different regions. Also, we found Heilongjiang, Shaanxi and Guizhou played important roles in HTNV evolution and migration. It is crucial to pay more attention on HFRS prevention and control in these areas. Furthermore, demographic history analyses of HTNV is relevant to rodents’ population, indicating the importance of avoiding contact between humans and host rodents. Meanwhile, the surveillance for rodent may be helpful, too. We discussed these in *Page 28, Line 487-488; Page 28, Line 490-495.*

Q7. English language should be revised and many typos and spelling mistakes corrected. Too many abbreviations are used and some are not defined (tMRCA lane 157, HPD lane 311).

A7. Thank you for pointing out the errors and we have corrected the issues.

Q8. Authors should define what they mean by analyses done from a holistic perspective (lanes26, 53 and 83) as compared to traditional approach (lane 57).

A8. Thank you for pointing it out. Previous studies usually focused on limited regions or time scale. In this study, we used all the available whole-genome sequences covers larger areas and time scale. Also, we inferred the evolution and spread history of HTNV with the help of with Bayesian stochastic search variable selection (BSSVS), which is rarely used for HTNV before. The explanations have been added in *Page 3, Line 50-54.*

Q9. At the end of the author summary, from lane 52, sentences are just redundant. Such repetition occurred several times as for instance in the discussion lanes 339-341.

A9. We are very sorry for our negligence and we have re-written the Abstract, Author summary and other parts to avoid redundant.

Q10. The nomenclature should be corrected by referring to the last ICTV recommendations with italic and majuscules used accordingly and should be harmonised along the manuscript. For instance HTNV does not belong to the Bunyaviridae family (lane 48) but to the Hantaviridae family in the genus Orthohantavirus as written lane 77.

A10. We are very sorry for our incorrectly writing. It has been corrected in the revised manuscript.

Q11. The colour code in Fig.4B defining the regions is different from the one in Fig.4A. This is confusing and this legend is just unnecessary and should be removed since the regions are clearly written on the left of the plot fig.4B. The authors could just draw black bars. In the case of Fig 5A the visualisation of the phylogeographic linkage could be better if diminishing the size of the character font.

A11. Thanks for suggestions. Figure 4B has been changed to a map colored by the root state posterior probability. The histogram of root was put on part A using the same color with the MCC tree *(Page 18, Line 297-304)*. Figure 5A was also re-drawn. The Bayes factor in Figure 5A has been presented as a color gradient now *(Page 19, Line315-321).*
