# Supplementary material for: Genetic diversity and evolution of Hantaan virus in China and its neighbors
Source: PLoS Negl Trop Dis. 2020 Aug 20;14(8):e0008090. doi: 10.1371/journal.pntd.0008090 (PMC7462299; doi:10.1371/journal.pntd.0008090)
Supplement: Attachment — Submitted filename: Response letter[PNTD-D-20-00046].docx [file pntd.0008090.s006.docx]

**Point to point responses**

**Reviewer 1：**

Q1. Given that I answered all these questions the first time around, I will ignore them this time. Just to say, I am happy with the way the authors have addressed the changes I requested, and am happy to now recommend this paper for publication. Below I will note some copy-editing changes that I noticed are required as I went back through the manuscript.

Line 85: "bio-information" should be "bioinformatic"
Line 189: "had higher number" should be "had a higher number"
Line 359: "others’ assumption" should be "others' assumptions"
Line 384/385: "Similar pattern" should be "In a similar pattern"
Line 389: "discuss too much here" should be "discuss them too much here" or similar
Line 400: "Furthermore, root state posterior probability of Guizhou was also in a high level." This is clunky. Something similar to "Furthermore, the posterior probability that Guizhou was the root location was high." would be better.

A1. Thank you so much for pointing out these errors in English usage. We have carefully checked the manuscript and corrected these errors in the text, and we hope you will find the English writing is improved in the revised manuscript. *(line85; line 193; line 367; line 392/393, line 397; line 408)*

**Reviewer 2：**

Q1.  The authors carried out the date randomisation test as requested, which revealed that the molecular clock rate of the dataset is potentially unreliable. This information should be presented before any discussion of the molecualr clock rate and phylogeography, as discussing results before revealing that they are unreliable may be misleading to readers. This would ideally be described on line 284. It would also be interesting for the authors to discuss the potential mechanism behind this unreliable clock rate.

A1. Thanks for the advice. The result of DRT has been presented before the discussion of the molecular clock rate and phylogeography analysis now *(line 287-290, page 18)*. Also, the paragraph about the potentially unreliable of molecular clock rate has been re-written *(line 454-466, page 26)*.

Q2. The authors also carried out the selection analysis as requested, including using the aBS-REL method. As mentioned, strong purifying selection can lead to an underestimation of branch lengths, however the authors have not fully investigated this. In order to determine whether their branch lengths are under estaimated the total branch length of the BEAST tree, and the tree produced by the aBS-REL method must be calculated in R using the ape package (http://ape- package.ird.fr/). If the total branch length for the BEAST tree is smaller than the aBS-REL tree, then the purifying selection in the dataset results in an underestimation of branch lengths and therefore a spurious substitution rate and TMRCA.

A2. Thanks for pointing out. We have calculated the total branch length of the MCC tree and aBSREL tree using the *compute.brlen()* function supplied by ape package in R (http://ape- package.ird.fr/). The total branch length of the MCC tree (8.604) is smaller than the aBSREL tree (14. 698), which indicating the underestimation of the substitution rate and tMRCA. We add these in *Discussion*(*line 462-466, page 26*.)

Q3. In figure S1, there are four outlier sequences isolated between 1980 and 1990 who's root-to-tip divergence does not match well with the tiem oftheir isolation. Have the authors tried excluding these fromt the analysis? These sequences may contribute to the unreliable clock rate.

A3. Thank you very much for the question. We tried excluding the four sequences before. But, as shown in the figure below, the result didn’t show stronger clock signal (Correlation Coefficient 0.2856; R squared 8.155E-2). Furthermore 3 of these 4 sequences isolated from Guizhou and the remaining one is strain 84Fli which is a classic isolate in China. In order to ensure enough sequences from Guizhou were included and we hope these 4 sequences could represent “older” strains before 2000, we still keep them in our dataset.

Fig1. Root-to-tip regression analyses using sequences after 2000**.**

**Reviewer 3：**

Q1.  I suggest that the authors correct a few inconsistencies in their manuscript:

- lane 190-192: since non explained abbreviations are used for the different hosts, in table 1 and figure 2, they should be introduced first after each species inside brackets.

- lane 194: the detail of the sequences are not appearing in fig 1 but in fig 2, also lane 197 the map shows a “geographic distribution” rather than ‘the localities of HTNV?”

- lane 234: it is written that isolates from Guizhou dispersed in group B,C, E, F and I, but neither N nor M sequences isolated from Guizhou province are found in group E as shown in fig 2.

- page 17: the new paragraph about natural selection is not clearly presented and it will be important to define in the text what is dN/dS used in the table 4.

- Lane 363: reassortment can occur between arthropod-borne viruses from the Bunyavirales order, but not only, since this has been described also for rodent-borne hantaviruses , as indicated in the references (44-48) mentioned by the authors.

- Lane 382: “ this finding may explain the high endemic(?) in these areas..” Do the authors mean “this finding might be explained by high-endemicity… or that this finding may explain the high level of epidemics in this area…” which is then redundant with the previous sentence (lane 381).

Also, there are still many spelling and syntax errors to be corrected in order to improve the writing.

A1. We are so grateful to you for pointing these out and we must apologize for our careless. All the inconsistencies have been modified.

- The abbreviations have been introduced after each species *(lane 194-196, page 10).*

- We have modified the sentence as “The sampling dates and geographic distribution of the sequences are shown in Fig 1.” *(lane 197-198, page 10)* The legend of Fig 1 was also modified as “Map showing the geographic distribution of Hantaan virus (HTNV) isolates included in this study.” *(lane 201-202, page 11)*

- As what you remined, isolates from Guizhou dispersed in group B, C, F and I. We have corrected the error here. *(lane 238, page 14)*

- The dN/dS has been defined in Method *(lane 140-144, page 8)*

- The inconsistency has been corrected in *lane 369-370, page 22*.

- The sentence has been modified as “This finding may explain the high level of epidemics in these areas…” *(lane 389-390, page 23)*. We want to express that the finding of Markov jump estimates can explain the epidemics in Heilongjiang and its surroundings.
